# Pattern of psychiatric hospitalization among women of childbearing age: a time series analysis, Brazil, 2012-2022

**DOI:** 10.1590/S2237-96222026v35e20250339.en

**Published:** 2026-06-07

**Authors:** Sofia Vezzani Kieling, Ingrid Araujo, Geórgia Osório, Fernanda Morandi Riegel Machado, Franciele Souza Santos, Lucas Primo de Carvalho Alves, Juliana Nichterwitz Scherer

**Affiliations:** ¹ Universidade do Vale do Rio dos Sinos, Divisão de Medicina, São Leopoldo, RS, Brazil Universidade do Vale do Rio dos Sinos Divisão de Medicina São Leopoldo RS Brazil

**Keywords:** Mental Disorders, Fertile Period, Hospitalization, Women’s Health, Time Series Studies, Trastornos Mentales, Periodo Fértil, Hospitalización, Salud de la Mujer, Estudios de Series Temporales.

## Abstract

**Objective:**

To analyze the trend and pattern of psychiatric hospitalizations among women aged 15 to 49 in Brazil, from 2012 to 2022, characterizing hospitalizations by macro-region, age group, race/skin color and reason for hospitalization.

**Methods:**

This is a time series analysis with data obtained from the Brazilian Unified Health System Department of Information Technology Hospital Morbidity System. The temporal trend of hospitalization rates was assessed using the Joinpoint Regression program, taking a 95% confidence interval (95%CI).

**Results:**

During the period, 677,608 psychiatric hospitalizations were recorded among women of childbearing age, corresponding to an average annual rate of 113 hospitalizations per 100,000 women. Between 2012 and 2016, an annual decrease of 5.1% in hospitalization rates was observed. The highest rates were identified in the Southern region (236.2/100,000), followed by the Northeast (169.9/100,000) and the Midwest (129.5/100,000). The 15-19 age group showed a significant upward trend, with an annual increase of 10.8% between 2015 and 2022. There was a significant increase in hospitalizations among women of Asian, mixed-race and Indigenous race/skin color, with 4.9% annual change between 2016 and 2022. The main reasons for hospitalization were mood disorders (38.1%) and schizophrenia, schizotypal disorders and delusional disorders (28.9%).

**Conclusion:**

There was an overall trend towards a decrease in psychiatric hospitalization rates, although there was an increase among younger women. Regional and racial disparities suggest difficulties in access to and continuity of mental health care. The predominance of psychotic disorders highlights gaps in care and the need for strategies focused on women’s mental health.

## Resumen

**Objetivo:**

Analizar la tendencia y el patrón de las hospitalizaciones psiquiátricas en mujeres de 15 a 49 años en Brasil, de 2012 a 2022, caracterizando las hospitalizaciones por macrorregiones, grupo de edad, raza/color de piel y motivo de hospitalización.

**Méto­dos:**

Análisis de series temporales con datos obtenidos del Sistema de Morbilidad Hospitalaria del Departamento de Informática del Sistema Único de Salud. La tendencia temporal de las tasas de hospitalización se evaluó mediante el programa *Joinpoint Regression*, considerando un intervalo de confianza del 95% (IC95%).

**Resultados:**

Durante el período, se registraron 677.608 hospitalizaciones psiquiátricas en mujeres en edad fértil, lo que corresponde a una tasa anual promedio de 113 hospitalizaciones por cada 100.000 mujeres. Entre 2012 y 2016, se observó una disminución anual del 5,1% en las tasas de hospitalización. Las tasas más altas se iden­tificaron en la región Sur (236,2/100.000), seguida del Nordeste (169,9/100.000) y el Centro-Oeste (129,5/100.000). El grupo de edad de 15 a 19 años mostró una importante tendencia al alza, con un incremento anual del 10,8% entre 2015 y 2022. Se observó un aumento significativo de las hospitalizaciones entre las mujeres de raza/color de piel amarillo, pardo e indígena, con una variación anual del 4,9 % entre 2016 y 2022. Los principales motivos de hospitalización fueron los trastornos del humor (38,1 %) y la esqui­zofrenia, los trastornos esquizotípicos y los trastornos delirantes (28,9 %).

**Conclusión:**

Se observó una tendencia a la disminución de las tasas de hospitalización psiquiátrica, pero un aumento entre las mujeres más jóvenes. Las disparidades regionales y raciales sugieren dificultades en el acceso y la continuidad de la atención de salud mental. El predominio de los trastornos psicóticos pone de relieve las deficiencias en la atención y la necesidad de estrategias centradas en la salud mental de las mujeres.

**Ethical aspects**

This research used public domain anonymized databases.

## Introduction 

Exposure to social, structural and economic determinants, as well as biological individualities, influence how each gender experiences and develops mental and behavioral disorders^1^. For example, women are at greater risk of developing mood disorders and are more likely to be hospitalized due to these disorders, with approximately double the risk of depressive and anxiety disorders^2^^,^^3^. Furthermore, it is known that the pattern and reason for hospitalization vary according to age group and stage of the reproductive cycle^4^.

In Brazil, in 2018-2019, most hospitalizations occurred among young women (mean age 28.7 years), with 32% of hospitalizations occurring among adolescents, 19% among pregnant adults, and 3% among pregnant adolescents^4^. Another Brazilian population-based study showed 31% prevalence of common mental disorders among women aged 20-50 years^5^. Although the literature points to higher occurrence of depressive disorders^6^, there are studies that alert as to the high prevalence of bipolar disorder and schizophrenia in women of childbearing age^7^. According to the 2022 Brazilian Demographic Census, 51% of the Brazilian population is female, 27% of whom are in the 15-49 age group^8^, therefore, investigating mental and behavioral disorders in women in this age group is important.

Although high prevalence of mental and behavioral disorders is indicated in the Brazilian female population^9^, recent data evaluating the epidemiological pattern of psychiatric hospitalization rates among women of childbearing age are lacking-this being a period in their lives in which hormonal and psychosocial factors can directly impact patterns of mental illness and psychiatric hospitalization. This study aims to analyze the trend and pattern of psychiatric hospitalizations among women aged 15 to 49 in Brazil, from 2012 to 2022, characterizing hospitalizations by macro-region, race/skin color, age group and reason for hospitalization.

## Methods 

Design

This is a time series analysis that assessed the occurrence of psychiatric hospitalizations among Brazilian women of childbearing age between 2012 and 2022.

Setting 

Brazil is the largest country in Latin America, and its territory is marked by wide ethnic and cultural diversity. Divided into five macro-regions (North, Northeast, South, Southeast and Midwest), its total population is around 203 million inhabitants. Of these, 51.5% are women, 26.7% of whom are of childbearing age^8^.

Participants

The study included women of childbearing age between 15 and 49 years (classification as per the World Health Organization) who were admitted to hospital due to disorders falling under Chapter V of the International Statistical Classification of Diseases and Related Health Problems (ICD-10), with the exception of reasons related to mental retardation (ICD-10 codes F70-79)^10^.

Variables

Hospitalization rates were stratified by Brazilian macro-regions (North, Northeast, South, Southeast, Midwest and Unknown/Overseas); age groups in years (15-19, 20-29, 30-39 and 40-49); race/skin color (White; Black; Asian, mixed-race and Indigenous; and unknown); and reason for hospitalization according to ICD-10 Chapter V - mental and behavioral disorders due to use of alcohol (F10); mental and behavioral disorders due to use of other psychoactive substances (F11-F19); schizophrenia, schizotypal and delusional disorders (F20-29); mood [affective] disorders (F30-39); neurotic, stress-related and somatoform disorders (F40-48); and other mental and behavioral disorders - behavioral syndromes associated with physiological disturbances and physical factors (F50-59).

Data sources and measurement

The data used in this study were extracted in March 2024 from the Brazilian Unified Health System Hospital Information System Hospital Morbidity Database, available online via the Brazilian Unified Health System Department of Information Technology Data Tabulation System. Additionally, we used data from the 2010 and 2022 Demographic Censuses, available from the Brazilian Institute of Geography and Statistics institutional website. National-level information was used, stratified by place of hospitalization.

Study size

The study included data on all women of childbearing age who were hospitalized for psychiatric reasons according to ICD-10 Chapter V in Brazil between 2012 and 2022.

Statistical methods 

The data collected from the Brazilian Unified Health System Department of Information Technology were compiled on an Excel for Windows spreadsheet. With regard to the Brazilian macro-regions, hospitalization rates were calculated per 100,000 female inhabitants aged 15 to 49 years, considering the 2010 census as the population base for the temporal analysis of the data between 2012-2016 and the 2022 census as a reference for 2017-2022, as they reflect, respectively, the most current demographic composition for each period analyzed. The remaining data were analyzed using absolute and relative frequencies.

Joinpoint regression analysis was used to assess temporal trends in psychiatric hospitalization rates. This method is widely employed in epidemiological time series studies. This approach allows for statistical identification of inflection points along the series, i.e. where a significant change occurs in the trend pattern.

Inflection points are not defined a priori by the authors, but rather are automatically estimated by the model through permutation tests. Each time segment delimited between two inflection points presents a specific annual percentage change (APC), which expresses the magnitude and direction of the trend in that interval. The presence of one or more APCs depends on the number of statistically significant inflection points detected in each stratified analysis.

Trends were classified as rising when APC had a positive value with a 95% confidence interval (95%CI) entirely above zero; falling when APC had a negative value with a 95% confidence interval entirely below zero; and stationary when the confidence interval included the value zero, regardless of the coefficient sign. Results with a p-value<0.050 were considered statistically significant.

Bias control

In order to ensure the uniformity and standardization of the data and calculated rates, only one information system was used. Control for serial autocorrelation and random fluctuations was performed using the Joinpoint Regression program. Stratified population analyses were conducted to assess differences in hospitalization patterns. Interpretation of the results took into consideration the possibility of external interferences resulting from changes in public policies and specific sociocultural and demographic characteristics of each macro-region.

## Results 

Between 2012 and 2022, 677,608 psychiatric hospitalizations of women of childbearing age were recorded in Brazil, representing an average annual rate of 113 hospitalizations per 100,000 women of childbearing age in the country. When assessing the temporal pattern of psychiatric hospitalization rates among women of childbearing age in Brazil during the period under analysis, a decrease of 5.1% per year (95%CI -13.7; -0.8) was identified between 2012 and 2016, followed by a stationary trend between 2017 and 2022 (APC 2.1%; 95%CI -0.6; 10.9) (Table 1).

Regarding the Brazilian macro-regions, the Southeast region had the highest absolute number of hospitalizations in the period 2012-2022 (270,558 cases), accounting for 39.9% of occurrences in the country, followed by the South and Northeast regions (200,679 and 113,771 cases, respectively) (Supplementary Table 1). When evaluating the hospitalization rates, it can be seen that, in the period 2012-2022, the Southern region had the highest average annual rates (236.17 hospitalizations per 100,000 women of childbearing age), followed by the Northeast region (169.95 hospitalizations per 100,000 women of childbearing age) and the Midwest region (129.49 per 100,000 women of childbearing age) (Supplementary Table 1).

When examining the temporal trends of hospitalization rates in the country’s macro-regions (Table 1), the Northern region, despite having the lowest absolute numbers, showed a growth pattern of 5.1% per year (95%CI 0.8; 9.8) in hospitalization numbers throughout the period analyzed. The Northeast region showed growth of 29.2% per year (95%CI 13.1; 80.4) until 2016, after which there was a stationary trend (APC 2%; 95%CI -22.7; 10.1) from 2016-2022. The Southeast region showed an annual decrease of 6.6% until 2016 (95%CI -13.7; -2.8) and an annual increase of 2.5% (95%CI 0.4; 7.9) in the number of hospitalizations between 2016 and 2022. In the Southern region, there was a stationary trend (APC 0.1%; 95%CI -2.2; 2.4) in psychiatric hospitalizations throughout the period analyzed. In the Midwest, there was a 6% annual decrease (95%CI -14.0; -2.1) in the hospitalization rate from 2012 to 2016 and then a stationary trend from 2016 to 2022 (APC 2%; 95%CI -0.3; 9.5). The variation in hospitalization rates between 2012 and 2022 in the country’s macro-regions is shown in Figure 1.

Between 2012 and 2022, the 30-39 age group had the highest number of hospitalizations due to psychiatric disorders in Brazil as a whole (Table 2). In the 15-19 age group, a 10.8% increase per year was observed (95%CI 7.3; 29.9) between 2015 and 2022. This is the largest percentage increase observed (Table 2). The 20-29 age group also showed growth between 2016 and 2022 (APC 5.8; 95%CI 2.9; 13.1). The 30-39 and 40-49 age groups showed a stationary trend in the last periods of the time series (2016-2022 and 2020-2022) (Table 2).

Regarding race/skin color, a higher absolute occurrence of hospitalizations was observed among White women. On the other hand, they showed a stationary trend throughout the period under analysis (APC -0.3; 95%CI -2.9; 2.3) (Table 2). A variation in the pattern of hospitalizations was observed among Black women, with a decrease between 2012-2016 (APC -9.1; 95%CI -19.5; -3.4), followed by a stationary trend between 2016-2022 (APC 3.3; 95%CI 0; 13.3). A falling trend was observed for Asian, mixed-race and Indigenous women between 2012-2016 (APC -4.6; 95%CI -12.9; -0.2), followed by a rising trend between 2016-2022 (APC 4.9; 95%CI 2.4; 11.9). Regarding patients with unknown race/skin color, a decrease in hospitalizations was observed between 2012-2014 (APC -13; 95%CI -19.6; -2.1), followed by a decreasing trend between 2014-2022 (APC -1.7; 95%CI -8.2; 7.5) (Supplementary Table 2).

In the period 2012-2022, the following reasons for hospitalization predominated: mood disorders (38.1%) and schizophrenia, schizotypal and delusional disorders (28.9%) (Table 2). It is noteworthy that there was a decrease in the temporal trends of hospitalizations for mental and behavioral disorders due to alcohol use and for schizophrenia, schizotypal and delusional disorders in the first half of the period (2012-2016), followed by a stationary trend in the subsequent segment (2016-2022) for almost all reasons for hospitalization (Table 2). Hospitalizations for mood disorders showed a stationary trend throughout the period (2012-2022) (APC 1.7; 95%CI -0.3; 3.6). Regional data stratified by age group, race/skin color and reason for hospitalization are shown in Supplementary Table 2.

**Table 1:** Annual percentage change (APC) and 95% confidence intervals (95%CI) of hospitalization rates due to psychiatric causes, by macro-region. Brazil, 2012-2022 (n=677,608)

Region	Absolute number of cases (2012-2022)	Average annual rate (per 100,000 inhabitants)	APC1^a^ (95%CI)	p-value	Lower year - Upper year	APC2^b^ (95%CI)	p-value	Lower year - Upper year
Brazil	677,608	113.5	-5.1 (-13.7; -0.8)	0.024	2012-2016	2.1 (-0.6; 10.9)	0.109	2016-2022
Macro-region								
North	28,760	52.8	5.1 (0.8; 9.8)	0.023	2012-2022			
Northeast	113,771	169.9	29.2^a^ (13.1; 80.4)	0.001	2012-2016	2.0 (-22.7; 10.1)	0.862	2016-2022
Midwest	63,840	129.5	-6.1^a^ (-14.0; -2.1)	0.005	2012-2016	2.1 (-0.3; 9.5)	0.090	2016-2022
Southeast	270,558	110.1	-6.6 (-13.7; -2.8)	<0.001	2012-2016	2.5 (0.4; 7.9)	0.024	2016-2022
South	200,679	236.2	0.1 (-2.2; 2.4)	0.963	2012-2022			

a Annual percentage change of the rate in the first time segment identified by the Joinpoint model; ^b^ annual percentage change of the rate in the following time segment identified by the Joinpoint model, when applicable.

**Table 2:** Annual percentage change (APC) and 95% confidence intervals (95%CI) of hospitalization rates due to psychiatric causes, by age group, race/color and reason for hospitalization. Brazil, 2012-2022 (n=677,608)

Variables	Absolute number of cases (2012-2022)	APC1^a^ (95%CI)	p-value	Lower year - Upper year	APC2^b^ (95%CI)	p-value	Lower year - Upper year
Age group							
15-19	55,874	-3.6 (-19.4; 6.1)	0.526	2012-2015	10.8 (7.3; 29.9)	0.007	2015-2022
20-29	165,414	-6.0 (-14.9; -1.1)	0.012	2012-2016	5.8 (2.9; 13.1)	0.001	2016-2022
30-39	220,661	-5.0 (-11.0; -2.2)	0.005	2012-2016	0.1 (-1.6; 6.0)	0.702	2016-2022
40-49	215,274	-3.7 (-7.7; - 2.5)	0.013	2012-2020	6.2 (-2.1; 11.6)	0.225	2020-2022
Race/color							
White	274,148	-0.3 (-2.9; 2.3)	0.773	2012-2022	-	-	-
Black	37,008	-9.1 (-19.5; -3.4)	0.001	2012-2016	3.3 (0; 13.3)	0.050	2016-2022
Asian, mixed-race and Indigenous	201,218	-4.6 (-12.9; -0.2)	0.040	2012-2016	4.9 (2.4; 11.9)	0.002	2016-2022
Unknown	165,297	-13.0 (-19.6; -2.1)	0.010	2012-2014	-1.7 (-8.2; 7.5)	0.605	2014-2022
Reason for hospitalization							
Mental and behavioral disorders due to use of alcohol	32,603	-8.1 (-14.8; -4.7)	<0.001	2012-2016	-1.4 (-3.4; 4.6)	0.527	2016-2022
Mental and behavioral disorders due to use of other psychoactive substances	88,929	-11.2 (-24.8; 0.2)	0.056	2012-2015	3.0 (-3.0; 19.6)	0.132	2015-2022
Schizophrenia. schizotypal and delusional disorders	215,034	-8.1 (-14.9; -4.6)	<0.001	2012-2016	-0.5 (-0.5; 4.9)	0.865	2016-2022
Mood [affective] disorders	253,907	1.7 (-0.1; 3.6)	0.071	2012-2022	-	-	-
Neurotic, stress-related and somatoform disorders	12,496	-18.0 (-29.4; 5.1)	0.174	2012-2014	6.8 (-3.0; 28.0)	0.056	2014-2022
Other disorders	55,293	2.2 (0.4; 4.6)	0.020	2012-2022	-	-	-

a Annual percentage change of the rate in the first time segment identified by the Joinpoint model; ^b^annual percentage change of the rate in the following time segment identified by the Joinpoint model, when applicable.

**Figure 1:**
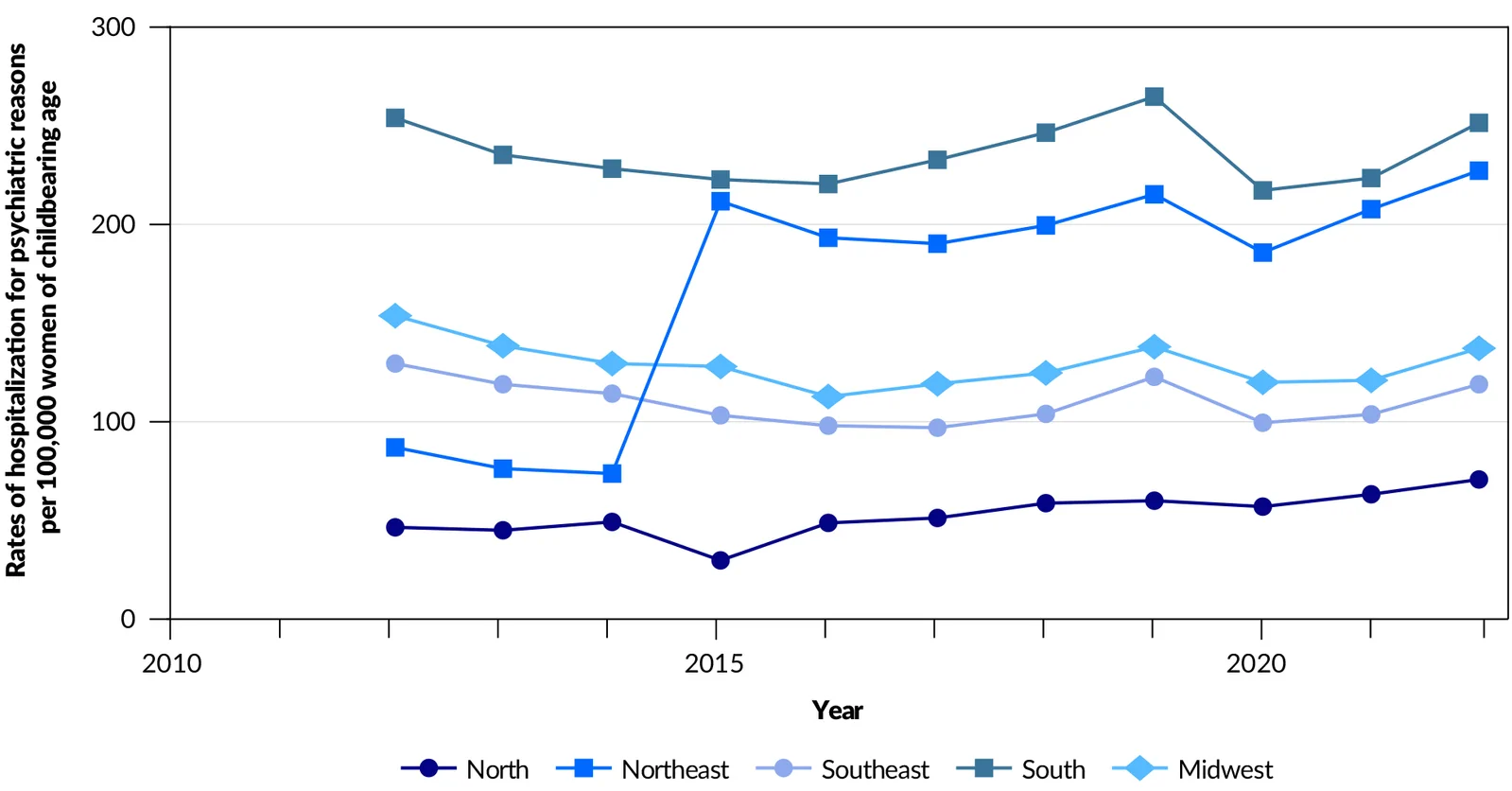
Rates of hospitalization for psychiatric reasons per 100,000 women of childbearing age, by macro-region. Brazil, 2012-2022 (n=677,608)

## Discussion

The results of this study show important changes in psychiatric hospitalizations among women of childbearing age in Brazil in the last decade (2012-2022). The decreasing trend observed between 2012 and 2016, followed by stability from 2016 to 2022, seems to reflect structural transformations in the psychosocial care network. The expansion of Psychosocial Care Centers, the initial strengthening of mental health actions in primary care and the encouragement of territorialized care may have contributed to initially reducing hospitalizations from 2012 to 2016. However, stabilization in the following years (2016-2022) suggests possible limits to this progress, especially given the underfunding of the Brazilian Unified Health System, the discontinuities in mental health policies and the gradual reintroduction of hospital-centered practices^11^. These factors, taken together, may have strained the problem-solving capacity of community services and created a scenario of increasing demand that is poorly absorbed by the out-of-hospital network.

In view of the findings observed, this study has limitations inherent to the use of secondary data, such as underreporting, inconsistencies in records and lack of diagnostic standardization. It is important to highlight that the hospitalization pattern characterized in our study does not reflect the psychiatric profile of the Brazilian population as a whole, since our analyses were restricted to women of childbearing age. Furthermore, hospitalization rates reflect local service availability and flows, not population prevalence, which limits extrapolation of the findings. Another relevant aspect to consider is the socioeconomic and cultural diversity between the Brazilian macro-regions, which results in variations in hospitalization protocols and heterogeneity in diagnostic criteria, these being factors that may limit the analysis of the true magnitude of mental and behavioral disorders in the population.

Even so, our results provide important elements for mental health planning. The differences observed between regions, age groups, race/skin color and reasons for hospitalization emphasize persistent inequalities and point to weaknesses in early detection and continuity of care in the territories.

The regional differences observed in our study follow a pattern consistent with the literature: the South and Southeast regions show a higher absolute number of hospitalizations, which may be related both to the higher prevalence of mental disorders in urbanized regions-marked by violence, inequality and economic stressors-and also to greater availability of specialized services, which favors greater case recording and access^12^. The Northeast region, in contrast, showed an inflection in the number of hospitalizations from 2014 onwards, with a considerable increase in these events. This finding may be associated with weaknesses and setbacks in the National Mental Health Policy, in the context of the implementation of the psychosocial care network deinstitutionalization policy that occurred in the same year. This setback contributed to greater use of psychiatric beds in hospital units^13^.

Hospitalizations were more frequent (40%) among White women nationally, however, distinct patterns emerge when analyzing by macro-region. In the North, Northeast and Midwest, in the period 2009-2018, mixed-race, Black, Asian and Indigenous women represented the largest proportion (60%) of hospitalizations, highlighting structural inequalities and discrepancies in access to care^14^. Although these regions concentrate the highest proportions of these racial groups in the Brazilian population (79%, 73% and 63%, respectively, according to the 2022 Census)^8^, these findings reinforce the presence of institutional racism in services, which hinders access to care, active outreach and continuity of treatment among Black women^14^^,^^15^. In addition, incompleteness of race/color records in these regions hinders accurate analysis of racial distribution and makes it difficult to develop effective public health policies. This set of barriers can lead to worsening of clinical conditions and, consequently, to greater risk of hospitalizations, especially in regions with less access to specialized services.

Regarding age, the results of this study reveal that, although hospitalizations are more frequently observed among women aged 30-39, the most significant increase occurred in the 15-19 age group. In the last decade, it has been observed that adolescents worldwide, of both sexes, are more exposed to school stress, sociocultural pressures, and changes in diagnosis that can increase identification of mental disorders^16^^,^^17^. In Spain (2000-2021) and Italy (2016-2023), an increase in hospitalizations for mood disorders in adolescents has been recorded over the last two decades^17^^,^^18^. This pattern has also been observed in the Brazilian context, as seen in this study.

In Finland, a study covering the period from 1996 to 2017 identified that hormonal changes related to the decline in estrogen during the perimenopause, among women with effect from the age of 30, may explain the greater severity of symptoms and relapses in psychotic disorders, favoring more frequent hospitalizations^19^. In the United States (2023-2024), it was observed that, although this generally begins after the age of 40, it can occur earlier in some women^20^. In Brazil, according to the 2022 Census, this distribution of hospitalizations may reflect the age structure of the Brazilian population, whereby the largest proportion of women (8%) are in the 35-39 age group^8^.

The concentration of hospitalizations in this age group seen in our results reaffirms the impact of psychosocial factors on the ability to reconcile work, affective and reproductive responsibilities. High family and professional demands have a significant impact on the mental health of women, but especially those aged between 35 and 39 years^19^^,^^20^.

Regarding the reasons for hospitalization, mood disorders (37%) and schizophrenia and related disorders (32%) predominated throughout the period analyzed in this study. The North (52 cases per 100,000 inhabitants) and Northeast (169 cases per 100,000 inhabitants) regions presented higher rates of hospitalizations for psychotic disorders, which may be a result of association of poverty and social inequality with higher risk and severity of these disorders^21^^-^^23^. Some of these hospitalizations may be associated with the "revolving door" phenomenon, marked by frequent readmissions due to the fragility of continued care, low adherence to treatment and difficulties in territorial follow-up^24^^-^^26^. An increase in hospitalizations due to substance use disorders, especially alcohol and illicit drugs, was also observed, indicating changes in the epidemiological profile and a pattern of growth in these hospitalizations in all regions of Brazil^17^^,^^27^.

Finally, the increase in hospitalizations among adolescents and the predominance of psychotic disorders help to highlight the need to strengthen psychosocial care networks, expand psychosocial care and organize more effective strategies for crisis prevention and management, especially for women of childbearing age-a group often made invisible in mental health policies. The results of this study provide important input for the planning and management of Brazilian Unified Health System services, corroborating the need for an individualized and targeted approach to mental health care for women of childbearing age.

## Data Availability

The data used in this study are derived from information that is publicly available from the Brazilian Unified Health System Department of Information Technology repository, specifically from the Hospital Information System - Hospital Morbidity database, available via the Data Tabulation System: [http://tabnet.datasus.gov. br/cgi/deftohtm.exe?sih/cnv/nibr.def]. The preprocessed dataset used for analysis, as well as the codes used for processing, are available upon request from the corresponding author.
