# Supplementary material for: Pattern of psychiatric hospitalization among women of childbearing age: a time series analysis, Brazil, 2012-2022
Source: Epidemiol Serv Saude. 2026 Jun 7;35:e20250339. doi: 10.1590/S2237-96222026v35e20250339.en (PMC13686375; doi:10.1590/S2237-96222026v35e20250339.en)
Supplement: Supplementary file 1 [file 2237-9622-ress-35-e20250339-sup-en.pdf]

Supplementary Table 1. Absolute numbers and rates of hospitalization for psychiatric reasons among women of childbearing age in Brazil, by Brazilian macro-regions. Brazil, 2012 – 2022 (n = 677,608)

Year	Indicator	Brazil	North	Northeast	Midwest	Southeast	South
2012	Total number	70,738	2,237	13,021	6,899	28,957	19,624
	Rate per 100,000 inhab.	130.36	46.61	87.25	153.93	129.66	254.05
2013	Total number	64,544	2,161	11,399	6,211	26,597	18,176
	Rate per 100,000 inhab.	118.95	45.03	76.38	138.58	119.09	235.30
2014	Total number	62,394	2,366	11,027	5,807	25,562	17,632
	Rate per 100,000 inhab.	114.98	49.30	73.89	129.56	114.46	228.26
2015	Total number	58,535	2,299	10,175	5,743	23,099	17,219
	Rate per 100,000 inhab.	107.87	29.76	212.00	128.14	103.43	222.91
2016	Total number	55,651	2,345	9,280	5,064	21,914	17,048
	Rate per 100,000 inhab.	102.56	48.86	193.35	112.99	98.12	220.70
2017	Total number	56,655	2,467	9,140	5,355	21,713	17,980
	Rate per 100,000 inhab.	104.41	51.40	190.44	119.48	97.22	232.76
2018	Total number	60,339	2,829	9,582	5,599	23,273	19,056
	Rate per 100,000 inhab.	111.20	58.94	199.64	124.92	104.21	246.69

2019	Número total	67,305	2,885	10,340	6,195	27,433	20,452
	Taxa a cada 100 mil hab.	124.03	60.11	215.44	138.22	122.84	264.76
2020	Total number	56,058	2,739	8,922	5,382	22,228	16,787
	Rate per 100,000 inhab.	103.31	57.07	185.89	120.08	99.53	217.32
2021	Total number	58,894	3,036	9,978	5,424	23,182	17,274
	Rate per 100,000 inhab.	108.53	63.26	207.90	121.02	103.80	223.62
2022	Total number	66,495	3,396	10,907	6,161	26,600	19,431
	Rate per 100,000 inhab.	122.54	70.76	227.25	137.46	119.11	251.55
Total	Total number	677,608	28,760	113,771	63,840	270,558	200,679
	Rate per 100,000 inhab,	113.52	52.83	169.95	129.49	110.13	236.17

Supplementary Table 2. Annual percentage change (APC) and 95% confidence intervals (95%CI) of rates of hospitalization for psychiatric reasons, by age group, race/skin color and diagnosis in the Brazilian macro-regions. Brazil, 2012 – 2022 (n = 677,608)

Region	Variables	Categories	APC1[b]	Lower year – upper year	APC2[c]	Lower year – Upper year
**North**	**Diagnosis**	Disorders due to alcohol use	-7.1[a]	2012-2020	46[a]	2020-2022
		Disorders due to other psychoactive substance use	33.6	2012-2014	-8.5[a]	2014-2022
		Schizoprenia, schizotypal and delusional disorders	1.9	2012-2020	13.1[a]	2020-2022
		Mood disorder	7.6[a]	2012-2022		
		Neurotic, stress-related and somatoform disorders	5.7	2012-2022		
		Other disorders	-13.2	2012-2014	12.3[a]	2014-2022
	**Age group (years)**	15 - 19	12.5[a]	2012-2022		
		20 - 29	1.1	2012-1017	8	2017-2022
		30 - 39	2.5[a]	2012-2022		
		40 - 49	3.8[a]	2012-2022		
	**Race/ skin color**	White	-11.9	2012-2016	34.7[a]	2016-2022
		Black	-5.9	2012-2017	24.9[a]	2017-2022
		Asian, mixed-race and Indigenous	1.6[a]	2012-2022		
		Unknown	5.7[a]	2012-2022		
**Northeast**	**Diagnosis**	Disorders due to alcohol use	-10.3[a]	2012-2015	1,4	2015-2022
		Disorders due to other psychoactive substance use	-11.9[a]	2012-2015	5[a]	2015-2022
		Schizoprenia, schizotypal and delusional disorders	-10.4[a]	2012-2017	0.4	2017-2022
		Mood disorder	-3.5	2012-2015	3.5[a]	2015-2022
		Neurotic, stress-related and somatoform disorders	-26.2[a]	2012-2014	9.5[a]	2014-2022
		Other disorders	-11.2	2012-2014	8.4[a]	2014-2022
	**Age group (years)**	15 - 19	-13.9	2012-2014	10.3[a]	2014-2022
		20 - 29	-9.5[a]	2012-2016	4[a]	2016-2022
		30 - 39	-7[a]	2012-2016	0.4	2016-2022
		40 - 49	-8[a]	2012-2017	2.1	2017-2022

	**Race/ skin color**	White	-11.3	2012-2015	5.4	2015-2022
		Black	-27.6[a]	2012-2017	5.3	2017-2022
		Asian, mixed-race and Indigenous	-7.4[a]	2012-2017	4.8[a]	2017-2022
		Unknown	-2.1[a]	2012-2022		
**Southeast**	**Diagnosis**	Disorders due to alcohol use	-8.9	2012-2017	-0.2	2017-2022
		Disorders due to other psychoactive substance use	-12.4[a]	2012-2016	2.9	2016-2022
		Schizoprenia, schizotypal and delusional disorders	-7.5[a]	2012-2016	-0.7	2016-2022
		Mood disorder	-6	2012-2015	5.4[a]	2015-2022
		Neurotic, stress-related and somatoform disorders	-13.6	2012-2014	5.7[a]	2014-2022
		Other disorders	-2.6	2012-2017	5.4	2017-2022
	**Age group (years)**	15 - 19	-6.8	2012-2015	10.1[a]	2015-2022
		20 - 29	-10.8[a]	2012-2015	5.2[a]	2015-2022
		30 - 39	-6.4[a]	2012-2016	0.8	2016-2022
		40 - 49	-4.5	2012-2020	9.5	2020-2022
	**Race/ skin color**	White	19.6	2012-2016	0.8	2016-2022
		Black	-8.2[a]	2012-2016	3.1	2016-2022
		Asian, mixed-race and Indigenous	-2.7	2012-2016	6.5	2016-2022
		Unknown	-8.8[a]	2012-2016	-0.7	2016-2022
**South**	**Diagnosis**	Disorders due to alcohol use	-5.3[a]	2012-2020	4.5	2020-2022
		Disorders due to other psychoactive substance use	-10.6	2012-2014	4.3[a]	2014-2022
		Schizoprenia, schizotypal and delusional disorders	-1.6	2012-2022		
		Mood disorder	0.3	2012-2022		
		Neurotic, stress-related and somatoform disorders	-3.5	2012-2022		
		Other disorders	2	2012-2022		
	**Age group (years)**	15 - 19	7.6[a]	2012-2022		
		20 - 29	-4.8	2012-2015	4.4[a]	2015-2022

		30 - 39	-1.5	2012-2022		
		40 - 49	-2.2	2012-2022		
	**Race/ skin color**	White	0.7	2012-2022		
		Black	-0.4	2012-2020	10.2	2020-2022
		Asian, mixed-race and Indigenous	3[a]	2012-2022		
		Unknown	-27.9[a]	2012-2014	-1.4	2014-2022
**Midwest**	**Diagnosis**	Disorders due to alcohol use	-3.3	2012-2022		
		Disorders due to other psychoactive substance use	-2	2012-2022		
		Schizoprenia, schizotypal and delusional disorders	-7.3[a]	2012-2020	4.3	2020-2022
		Mood disorder	-2.3	2012-2016	3.5[a]	2016-2022
		Neurotic, stress-related and somatoform disorders	-16.6	2012-2015	22.7[a]	2015-2022
		Other disorders	-10	2012-2014	7.2	2014-2022
	**Faixa etária (anos)**	15 - 19	-12	2012-2014	12.1[a]	2014-2022
		20 - 29	-7.7[a]	2012-2016	7.9[a]	2016-2022
		30 - 39	-2.7[a]	2012-2022		
		40 - 49	-3.1[a]	2012-2022		
	**Race/ skin color**	White	-1.9	2012-2018	15.3[a]	2018-2022
		Black	-16.5[a]	2012-2015	10.3[a]	2015-2022
		Asian, mixed-race and Indigenous	-2.3[a]	2012-2020	34.2[a]	2020-2022
		Unknown	-2.2	2012-2020	-20.3[a]	2020-2022

[a] indicates statistical significance; [b] annual percentage change of the rate in the first time segment identified by the Joinpoint model; [c] annual percentage change of the rate in the subsequent time segment identified by the Joinpoint model, when applicable.
