# Supplementary material for: Pattern of psychiatric hospitalization among women of childbearing age: a time series analysis, Brazil, 2012-2022
Source: Epidemiol Serv Saude. 2026 Jun 7;35:e20250339. doi: 10.1590/S2237-96222026v35e20250339.en (PMC13686375; doi:10.1590/S2237-96222026v35e20250339.en)
Supplement: Supplementary file 2 [file 2237-9622-ress-35-e20250339-sup-pt.pdf]

Tabela Suplementar 1. Números absolutos e taxas de internações por causas psiquiátricas entre mulheres em idade fértil no Brasil segundo as macrorregiões brasileiras. Brasil, 2012 – 2022 (n = 677.608)

Ano	Indicador	Brasil	Norte	Nordeste	Centro-Oeste	Sudeste	Sul
2012	Número total	70.738	2.237	13.021	6.899	28.957	19.624
	Taxa a cada 100 mil hab.	130,36	46,61	87,25	153,93	129,66	254,05
2013	Número total	64.544	2.161	11.399	6.211	26.597	18.176
	Taxa a cada 100 mil hab.	118,95	45,03	76,38	138,58	119,09	235,30
2014	Número total	62.394	2.366	11.027	5.807	25.562	17.632
	Taxa a cada 100 mil hab.	114,98	49,30	73,89	129,56	114,46	228,26
2015	Número total	58.535	2.299	10.175	5.743	23.099	17.219
	Taxa a cada 100 mil hab.	107,87	29,76	212,00	128,14	103,43	222,91
2016	Número total	55.651	2.345	9.280	5.064	21.914	17.048
	Taxa a cada 100 mil hab.	102,56	48,86	193,35	112,99	98,12	220,70
2017	Número total	56.655	2.467	9.140	5.355	21.713	17.980
	Taxa a cada 100 mil hab.	104,41	51,40	190,44	119,48	97,22	232,76
2018	Número total	60.339	2.829	9.582	5.599	23.273	19.056
	Taxa a cada 100 mil hab.	111,20	58,94	199,64	124,92	104,21	246,69
2019	Número total	67.305	2.885	10.340	6.195	27.433	20.452
	Taxa a cada 100 mil hab.	124,03	60,11	215,44	138,22	122,84	264,76

2020	Número total	56.058	2.739	8.922	5.382	22.228	16.787
	Taxa a cada 100 mil hab.	103,31	57,07	185,89	120,08	99,53	217,32
2021	Número total	58.894	3.036	9.978	5.424	23.182	17.274
	Taxa a cada 100 mil hab.	108,53	63,26	207,90	121,02	103,80	223,62
2022	Número total	66.495	3.396	10.907	6.161	26.600	19.431
	Taxa a cada 100 mil hab.	122,54	70,76	227,25	137,46	119,11	251,55
Total	Número total	677.608	28.760	113.771	63.840	270.558	200.679
	Taxa a cada 100 mil hab.	113,52	52,83	169,95	129,49	110,13	236,17

Tabela Suplementar 2. Variação percentual anual (VPA) e intervalos de confiança de 95% (IC95%) das taxas de internação por causas psiquiátricas segundo faixa etária, raça/cor da pele e diagnóstico nas macrorregiões brasileiras. Brasil, 2012 – 2022 (n = 677.608)

Região	Variáveis	Categorias	VPA1[b]	Ano inferior – Ano superior	VPA2[c]	Ano inferior – Ano superior
**Norte**	**Diagnóstico**	Transtorno por uso de álcool	-7,1[a]	2012-2020	46[a]	2020-2022
		Transtorno por uso de outras substâncias psicoativas	33,6	2012-2014	-8,5[a]	2014-2022
		Esquizofrenia, transtornos esquizotípicos e transtornos delirantes	1,9	2012-2020	13,1[a]	2020-2022
		Transtorno de humor afetivo	7,6[a]	2012-2022		
		Transtornos neuróticos, relacionados ao "stress" e somatoformes	5,7	2012-2022		
		Outros transtornos	-13,2	2012-2014	12,3[a]	2014-2022
	**Faixa etária (anos)**	15 - 19	12,5[a]	2012-2022		
		20 - 29	1,1	2012-1017	8	2017-2022
		30 - 39	2,5[a]	2012-2022		
		40 - 49	3,8[a]	2012-2022		
	**Raça/ cor da pele**	Branca	-11,9	2012-2016	34,7[a]	2016-2022
		Preta	-5,9	2012-2017	24,9[a]	2017-2022
		Amarela, Parda e Indígena	1,6[a]	2012-2022		
		Ignorada	5,7[a]	2012-2022		
**Nordeste**	**Diagnóstico**	Transtorno por uso de álcool	-10,3[a]	2012-2015	1,4	2015-2022
		Transtorno por uso de outras substâncias psicoativas	-11,9[a]	2012-2015	5[a]	2015-2022
		Esquizofrenia, transtornos esquizotípicos e transtornos delirantes	-10,4[a]	2012-2017	0,4	2017-2022
		Transtorno de humor afetivo	-3,5	2012-2015	3,5[a]	2015-2022
		Transtornos neuróticos, relacionados ao "stress" e somatoformes	-26,2[a]	2012-2014	9,5[a]	2014-2022
		Outros transtornos	-11,2	2012-2014	8,4[a]	2014-2022
	**Faixa etária (anos)**	15 - 19	-13,9	2012-2014	10,3[a]	2014-2022
		20 - 29	-9,5[a]	2012-2016	4[a]	2016-2022

		30 - 39	-7[a]	2012-2016	0,4	2016-2022
		40 - 49	-8[a]	2012-2017	2,1	2017-2022
	**Raça/ cor da pele**	Branca	-11,3	2012-2015	5,4	2015-2022
		Preta	-27,6[a]	2012-2017	5,3	2017-2022
		Amarela, Parda e Indígena	-7,4[a]	2012-2017	4,8[a]	2017-2022
		Ignorada	-2,1[a]	2012-2022		
**Sudeste**	**Diagnóstico**	Transtorno por uso de álcool	-8,9	2012-2017	-0,2	2017-2022
		Transtorno por uso de outras substâncias psicoativas	-12,4[a]	2012-2016	2,9	2016-2022
		Esquizofrenia, transtornos esquizotípicos e transtornos delirantes	-7,5[a]	2012-2016	-0,7	2016-2022
		Transtorno de humor afetivo	-6	2012-2015	5,4[a]	2015-2022
		Transtornos neuróticos, relacionados ao "stress" e somatoformes	-13,6	2012-2014	5,7[a]	2014-2022
		Outros transtornos	-2,6	2012-2017	5,4	2017-2022
	**Faixa etária (anos)**	15 - 19	-6,8	2012-2015	10,1[a]	2015-2022
		20 - 29	-10,8[a]	2012-2015	5,2[a]	2015-2022
		30 - 39	-6,4[a]	2012-2016	0,8	2016-2022
		40 - 49	-4,5	2012-2020	9,5	2020-2022
	**Raça/ cor da pele**	Branca	19,6	2012-2016	0,8	2016-2022
		Preta	-8,2[a]	2012-2016	3,1	2016-2022
		Amarela, Parda e Indígena	-2,7	2012-2016	6,5	2016-2022
		Ignorada	-8,8[a]	2012-2016	-0,7	2016-2022
**Sul**	**Diagnóstico**	Transtorno por uso de álcool	-5,3[a]	2012-2020	4,5	2020-2022
		Transtorno por uso de outras substâncias psicoativas	-10,6	2012-2014	4,3[a]	2014-2022
		Esquizofrenia, transtornos esquizotípicos e transtornos delirantes	-1,6	2012-2022		
		Transtorno de humor afetivo	0,3	2012-2022		

		Transtornos neuróticos, relacionados ao "stress" e somatoformes	-3,5	2012-2022		
		Outros transtornos	2	2012-2022		
	**Faixa etária (anos)**	15 - 19	7,6[a]	2012-2022		
		20 - 29	-4,8	2012-2015	4,4[a]	2015-2022
		30 - 39	-1,5	2012-2022		
		40 - 49	-2,2	2012-2022		
	**Raça/ cor da pele**	Branca	0,7	2012-2022		
		Preta	-0,4	2012-2020	10,2	2020-2022
		Amarela, Parda e Indígena	3[a]	2012-2022		
		Ignorada	-27,9[a]	2012-2014	-1,4	2014-2022
**Centro-Oeste**	**Diagnóstico**	Transtorno por uso de álcool	-3,3	2012-2022		
		Transtorno por uso de outras substâncias psicoativas	-2	2012-2022		
		Esquizofrenia, transtornos esquizotípicos e transtornos delirantes	-7,3[a]	2012-2020	4,3	2020-2022
		Transtorno de humor afetivo	-2,3	2012-2016	3,5[a]	2016-2022
		Transtornos neuróticos, relacionados ao "stress" e somatoformes	-16,6	2012-2015	22,7[a]	2015-2022
		Outros transtornos	-10	2012-2014	7,2	2014-2022
	**Faixa etária (anos)**	15 - 19	-12	2012-2014	12,1[a]	2014-2022
		20 - 29	-7,7[a]	2012-2016	7,9[a]	2016-2022
		30 - 39	-2,7[a]	2012-2022		
		40 - 49	-3,1[a]	2012-2022		
	**Raça/ cor da pele**	Branca	-1,9	2012-2018	15,3[a]	2018-2022
		Preta	-16,5[a]	2012-2015	10,3[a]	2015-2022
		Amarela, Parda e Indígena	-2,3[a]	2012-2020	34,2[a]	2020-2022
		Ignorada	-2,2	2012-2020	-20,3[a]	2020-2022

[a] indica significância estatística; [b] variação percentual anual da taxa no primeiro segmento temporal identificado pelo modelo Joinpoint; [c] variação percentual anual da taxa no segmento temporal subsequente identificado pelo modelo Joinpoint, quando aplicável.
