# Supplementary material for: Pattern of psychiatric hospitalization among women of childbearing age: a time series analysis, Brazil, 2012-2022
Source: Epidemiol Serv Saude. 2026 Jun 7;35:e20250339. doi: 10.1590/S2237-96222026v35e20250339.en (PMC13686375; doi:10.1590/S2237-96222026v35e20250339.en)
Supplement: Translation

# Translation: Padrão de internação psiquiátrica entre mulheres em idade fértil: análise de série temporal, Brasil, 2012-2022

**DOI:** 10.1590/S2237-96222026v35e20250339.pt

**Authors:** Sofia Vezzani Kieling, Ingrid Araujo, Geórgia Osório, Fernanda Morandi Riegel Machado, Franciele Souza Santos, Lucas Primo de Carvalho Alves, Juliana Nichterwitz Scherer

**Affiliations:** ¹ Universidade do Vale do Rio dos Sinos, Divisão de Medicina, São Leopoldo, RS, Brasil Universidade do Vale do Rio dos Sinos Divisão de Medicina São Leopoldo RS Brasil

**Keywords:** Transtornos Mentais, Período Fértil, Hospitalização, Saúde da Mulher, Estudos de Séries Temporais

## Abstract

**Objetivo:**

Analisar a tendência e o padrão das internações psiquiátricas entre mulheres de 15 a 49 anos no Brasil, no período 2012- 2022, caracterizando as internações por macrorregiões, faixa etária, raça/cor da pele e motivo de internação.

**Métodos:**

Análise de série temporal com dados obtidos do Sistema de Morbidade Hospitalar do Departamento de Informática do Sistema Único de Saúde. A tendência temporal das taxas de internação foi avaliada pelo programa Joinpoint Regression, considerando um intervalo de confiança de 95% (IC95%).

**Resultados:**

No período, foram registradas 677.608 internações psiquiátricas entre mulheres em idade fértil, o que corresponde a uma taxa média anual de 113 internações por 100 mil mulheres. Entre 2012-2016, observou-se decréscimo anual de 5,1% nas taxas de internação. As maiores taxas foram identificadas na região Sul (236,2/100 mil), seguida do Nordeste (169,9/100 mil) e Centro-Oeste (129,5/100 mil). A faixa etária de 15 a 19 anos apresentou tendência crescente significativa, com aumento anual de 10,8% entre 2015-2022. Houve crescimento significativo das hospitalizações entre mulheres de raça/cor da pele amarela, parda e indígena, com variação anual de 4,9% entre 2016-2022. Os principais motivos de internação foram transtornos de humor (38,1%) e esquizofrenia, transtornos esquizotípicos e transtornos delirantes (28,9%).

**Conclusão:**

Houve tendência a decréscimo nas taxas de internação psiquiátricas, mas crescimento entre as mulheres mais jovens. Disparidades regionais e raciais sugerem dificuldades de acesso e de continuidade do cuidado à saúde mental. A predominância de transtornos psicóticos destaca lacunas assistenciais e a necessidade de estratégias voltadas à saúde mental feminina.

**Aspectos éticos**

Esta pesquisa utilizou bancos de dados de domínio público e anonimizados.

## Introdução 

A exposição a determinantes sociais, estruturais e econômicos, assim como individualidades biológicas, influenciam na maneira como cada gênero experiencia e desenvolve transtornos mentais e comportamentais^1^. Por exemplo, mulheres apresentam maiores riscos de desenvolver transtornos de humor e maior chance de internação hospitalar por esses transtornos, com risco aproximadamente 2 vezes maior para transtornos depressivos e de ansiedade^2^^,^^3^. Além disso, sabe-se que o padrão e o motivo de internação variam conforme a faixa etária e a etapa do ciclo de reprodução^4^.

No Brasil, em 2018-2019, a maior parte das internações ocorreu em mulheres jovens (média de idade de 28,7 anos), sendo que 32% das internações ocorreram entre adolescentes, 19% entre adultas gestantes e 3% entre adolescentes gestantes^4^. Outro estudo brasileiro de base populacional mostrou uma prevalência de transtornos mentais comuns de 31% entre mulheres entre 20-50 anos^5^. Apesar de a literatura apontar a maior ocorrência de transtornos depressivos^6^, há estudos que alertam para a alta prevalência de transtorno bipolar e esquizofrenia em mulheres em idade fértil^7^. De acordo com o Censo Demográfico Brasileiro de 2022, 51% da população brasileira é do sexo feminino e 27% dessas estão na faixa etária de 15-49 anos^8^, sendo, portanto, importante a investigação de transtornos mentais e comportamentais em mulheres nesta faixa etária.

Ainda que se indique uma alta prevalência de transtornos mentais e comportamentais na população brasileira feminina^9^, faltam dados recentes que avaliem o padrão epidemiológico nas taxas de internações psiquiátricas entre as mulheres em idade fértil - período em que fatores hormonais e psicossociais podem repercutir diretamente nos padrões de adoecimento mental e internação psiquiátrica. O presente estudo tem como objetivo analisar a tendência e o padrão das internações psiquiátricas entre mulheres de 15 a 49 anos no Brasil, no período 2012-2022, caracterizando as internações por macrorregiões, raça/cor da pele, faixa etária e motivo de internação.

## Métodos 

Delineamento

Trata-se de uma análise de série temporal que avaliou ocorrências de internações psiquiátricas da população brasileira feminina em idade fértil entre o período de 2012-2022. 

Contexto 

O Brasil é o maior país da América Latina, e seu território é marcado por uma ampla diversidade étnica e cultural. Dividido em cinco macrorregiões (Norte, Nordeste, Sul, Sudeste e Centro-Oeste), sua população totaliza cerca de 203 mil habitantes. Destes, 51,5% são mulheres, das quais 26,7% encontram-se em idade fértil^8^. 

Participantes

Participaram do estudo mulheres em idade fértil entre 15 e 49 anos (classificação conforme a Organização Mundial de Saúde) que foram internadas em unidade hospitalar por motivo de transtornos do capítulo V da Classificação Estatística Internacional de Doenças e Problemas Relacionados à Saúde (CID-10), com exceção de motivos relacionados ao retardo mental (CID-10 F70 - 79)^10^.

Variáveis do estudo

As taxas de internações foram estratificadas por território brasileiro em macrorregiões (Norte, Nordeste, Sul, Sudeste, Centro-Oeste e Ignorado/Exterior); faixas etárias (15-19 anos, 20-29 anos, 30-39 anos, e 40-49 anos); raça/cor da pele (brancas; pretas; amarelas, pardas e indígenas; e ignorado); e motivo de internação conforme capítulo V do CID-10 - transtornos mentais e comportamentais devido ao uso de álcool (F10); transtornos mentais e comportamentais devido ao uso de outras substâncias psicoativas (F11-F19); esquizofrenia, transtornos esquizotípicos e transtornos delirantes (F20-29); transtorno de humor afetivo (F30-39); transtornos neuróticos, relacionados ao estresse e somatoformes (F40-48); e outros transtornos mentais e comportamentais - síndromes comportamentais associadas a disfunções fisiológicas e a fatores de risco (F50-59).

Fontes de dados e mensuração

Os dados utilizados foram extraídos em março de 2024 a partir da base de dados de Morbidade Hospitalar do Sistema Único de Saúde - Sistema de Informações Hospitalares, disponíveis no Sistema de Tabulação de Dados do site do Departamento de Informática do Sistema Único de Saúde. Adicionalmente, foram utilizados dados dos Censos Demográficos de 2010 e de 2022, disponibilizados pelo Instituto Brasileiro de Geografia e Estatística em seu site institucional. As informações foram consideradas em nível nacional, com estratificação por local de internação. 

Tamanho do estudo

O estudo abrangeu dados de todas as mulheres em idade fértil que internaram em unidade hospitalar por motivo psiquiátrico segundo o capítulo V do CID-10 em território brasileiro entre 2012-2022. 

Métodos estatísticos

Os dados coletados do Departamento de Informática do Sistema Único de Saúde foram reunidos em uma planilha do programa Excel para Windows. Para as macrorregiões brasileiras, foram calculadas as taxas de internação para cada 100 mil habitantes do sexo feminino na faixa etária entre 15 e 49 anos, considerando o censo de 2010 como base populacional para a análise temporal dos dados entre 2012-2016 e o censo de 2022 como referência para 2017-2022, por refletirem, respectivamente, a composição demográfica mais atual para cada período analisado. Os demais dados foram analisados com frequências absoluta e relativa. 

Para avaliar as tendências temporais das taxas de internação psiquiátrica, foi utilizada a análise de regressão por pontos de junção (joinpoint regression), método amplamente empregado em estudos epidemiológicos de séries temporais. Essa abordagem permite identificar, de forma estatística, pontos de inflexão ao longo da série, nos quais ocorre mudança significativa no padrão da tendência. 

Os pontos de junção não são definidos a priori pelos autores, sendo estimados automaticamente pelo modelo por meio de testes de permutação. Cada segmento temporal delimitado entre dois pontos de junção apresenta uma variação percentual anual (VPA) específica, que expressa a magnitude e a direção da tendência naquele intervalo. A presença de uma ou mais VPAs depende do número de pontos de inflexão estatisticamente significativos detectados em cada análise estratificada. 

As tendências foram classificadas como de aumento quando a VPA apresentou valor positivo com intervalo de confiança de 95% (IC95%) inteiramente acima de zero; de redução quando a VPA apresentou valor negativo com intervalo de confiança de 95% inteiramente abaixo de zero; e estacionária quando o intervalo de confiança incluiu o valor zero, independentemente do sinal do coeficiente. Foram considerados estatisticamente significativos os resultados que apresentaram um p-valor*<*0,050. 

Controle de vieses

Visando garantir a uniformidade e a padronização dos dados e das taxas calculadas, utilizou-se exclusivamente um único sistema de informação. O controle da autocorrelação serial e das flutuações aleatórias foi realizado por meio do programa Joinpoint Regression. Foram realizadas análises estratificadas da população para avaliar as diferenças nos padrões de internação. Na interpretação dos resultados, considerou-se a possibilidade de interferências externas decorrentes de alterações nas políticas públicas e características socioculturais e demográficas específicas de cada macrorregião.

## Resultados 

No período 2012-2022, foram registradas 677.608 internações psiquiátricas de mulheres em idade fértil no Brasil, o que representa uma taxa média anual de 113 internações a cada 100 mil mulheres em idade fértil no país. Ao avaliar o padrão temporal das taxas de internação psiquiátrica entre mulheres em idade fértil no Brasil no período em análise, identificou-se um decréscimo de 5,1% ao ano (IC95% -13,7; -0,8) entre 2012-2016, seguido por uma tendência estacionária entre 2017-2022 (VPA 2,1%; IC95% -0,6; 10,9) (Tabela 1).

Em relação às macrorregiões, o Sudeste é a região com o maior número absoluto de internações no período 2012-2022 (270.558 casos), representando 39,9% das ocorrências no país, seguido pelas regiões Sul e Nordeste (200.679 e 113.771 casos) (Tabela Suplementar 1). Ao avaliarmos as taxas de internação, observa-se que, no período de 2012-2022, a região Sul apresentou as maiores taxas médias anuais (236,17 internações a cada 100 mil mulheres em idade fértil), seguida pela região Nordeste (169,95 internações a cada 100 mil mulheres em idade fértil) e Centro-Oeste (129,49 a cada 100 mil mulheres em idade fértil) (Tabela Suplementar 1). 

Observando as tendências temporais das taxas de internações nas macrorregiões do país (Tabela 1), a região Norte, apesar de ter os menores números absolutos, apresentou um padrão de crescimento de 5,1% ao ano (IC95% 0,8; 9,8) nos números de internações durante todo o período de análise. A região Nordeste demonstrou crescimento de 29,2% ao ano (IC95% 13,1; 80,4) até 2016, depois houve tendência estacionária (VPA 2%; IC95% -22,7: 10,1) de 2016-2022. A região Sudeste apresentou decréscimo anual de 6,6% até 2016 (IC95% -13,7; -2,8) e crescimento de 2,5% ao ano (IC95% 0,4; 7,9) no número de internações entre 2016-2022 Na região Sul houve tendência estacionária (VPA 0,1%; IC95% -2,2; 2,4) nas internações psiquiátricas durante todo o período analisado. No Centro-Oeste houve decréscimo de 6% ao ano (IC95% -14,0; -2,1) na taxa de internação de 2012-2016 e, depois, tendência estacionária de 2016 a 2022 (VPA 2%; IC95% -0,3; 9,5). A variação nas taxas de internação entre 2012-2022 nas macrorregiões do país está representada na Figura 1.

Entre 2012-2022, a faixa etária que mais teve internações por transtornos psiquiátricos no país foi 30-39 anos (Tabela 2). Na faixa etária 15-19 anos, observou-se crescimento de 10,8% ao ano (IC95% 7,3; 29,9) entre 2015-2022. É o maior aumento percentual observado (Tabela 2). A faixa etária 20-29 anos também apresentou crescimento entre 2016-2022 (VPA 5,8; IC95% 2,9; 13,1). As faixas etárias 30-39 e 40-49 apresentaram tendência estacionária nos últimos períodos da série temporal (2016-2022 e 2020-2022) (Tabela 2).

Em relação à raça/cor da pele, observou-se maior ocorrência absoluta de internações entre mulheres brancas. Por outro lado, houve tendência estacionária durante todo o período em análise (VPA -0,3; IC95% -2,9; 2,3) (Tabela 2). Observou-se variação do padrão de internações em mulheres pretas, com decréscimo entre 2012-2016 (VPA -9,1; IC95% -19,5; -3,4), seguido por tendência estacionária entre 2016-2022 (VPA 3,3; IC95% 0; 13,3). Para mulheres amarelas, pardas e indígenas, observou-se tendência de decréscimo entre 2012-2016 (VPA -4,6; IC95% -12,9; -0,2), seguida por tendência de crescimento entre 2016-2022 (VPA 4,9; IC95% 2,4;11,9). Em relação às pacientes com raça/cor de pele ignorada, observou-se decréscimo nas internações entre 2012-2014 (VPA -13; IC95% -19,6; -2,1), seguido por tendência ao decréscimo entre 2014-2022 (VPA -1,7: IC95% -8,2; 7,5) (Tabela Suplementar 2). 

No período de 2012-2022, predominaram os seguintes motivos para internação: transtornos de humor (38,1%) e esquizofrenia, transtornos esquizotípicos e transtornos delirantes (28,9%) (Tabela 2). Salienta-se que houve decréscimo nas tendências temporais das internações por transtornos mentais e comportamentais devido ao uso de álcool e por esquizofrenia, transtornos esquizotípicos e transtornos delirantes na primeira metade do período (2012-2016), seguido por tendência estacionária no segmento subsequente (2016-2022) para quase todos os motivos de internação (Tabela 2). Internações por transtornos de humor apresentaram tendência estacionária durante todo o período (2012-2022) (VPA 1,7; IC95% -0,3; 3,6). Os dados regionais estratificados por faixa etária, raça/cor de pele e motivo de internação estão apresentados na Tabela Suplementar 2. 

**Tabela 1:** Variação percentual anual (VPA) e intervalos de confiança de 95% (IC95%) das taxas de internação por causas psiquiátricas nas macrorregiões. Brasil, 2012-2022 (n=677.608)

Região	Número absoluto de casos (2012-2022)	Taxa anual média (a cada 100 mil habitantes)	VPA1^a^ (IC95%)	p-valor	Ano inferior - Ano superior	VPA2^b^ (IC95%)	p-valor	Ano inferior - Ano superior
Brasil	677.608	113,5	-5,1 (-13,7; -0,8)	0,024	2012-2016	2,1 (-0,6; 10,9)	0,109	2016-2022
Macrorregião								
Norte	28.760	52,8	5,1 (0,8; 9,8)	0,023	2012-2022			
Nordeste	113.771	169,9	29,2^a^ (13,1; 80,4)	0,001	2012-2016	2,0 (-22,7; 10,1)	0,862	2016-2022
Centro-Oeste	63.840	129,5	-6,1^a^ (-14,0; -2,1)	0,005	2012-2016	2,1 (-0,3; 9,5)	0,090	2016-2022
Sudeste	270.558	110,1	-6,6 (-13,7; -2,8)	<0,001	2012-2016	2,5 (0,4; 7,9)	0,024	2016-2022
Sul	200.679	236,2	0,1 (-2,2; 2,4)	0,963	2012-2022			

a variação percentual anual da taxa no primeiro segmento temporal identificado pelo modelo Joinpoint; ^b^ variação percentual anual da taxa no segmento temporal subsequente identificado pelo modelo Joinpoint, quando aplicável.

**Tabela 2:** Variação percentual anual (VPA) e intervalos de confiança de 95% (IC95%) das taxas de internação por causas psiquiátricas segundo faixa etária, raça/cor e motivo de internação. Brasil, 2012-2022 (n=677.608)

Variáveis	Número absoluto de casos (2012-2022)	VPA1^a^ (IC95%)	p-valor	Ano inferior - Ano superior	VPA2^b^ (IC95%)	p-valor	Ano inferior - Ano superior
Anos							
15-19	55.874	-3,6 (-19,4; 6,1)	0,526	2012-2015	10,8 (7,3; 29,9)	0,007	2015-2022
20-29	165.414	-6,0 (-14,9; -1,1)	0,012	2012-2016	5,8 (2,9; 13,1)	0,001	2016-2022
30-39	220.661	-5,0 (-11,0; -2,2)	0,005	2012-2016	0,1 (-1,6; 6,0)	0,702	2016-2022
40-49	215.274	-3,7 (-7,7; - 2,5)	0,013	2012-2020	6,2 (-2,1; 11,6)	0,225	2020-2022
Raça/cor							
Branca	274.148	-0,3 (-2,9; 2,3)	0,773	2012-2022	-	-	-
Preta	37.008	-9,1 (-19,5; -3,4)	0,001	2012-2016	3,3 (0; 13,3)	0,050	2016-2022
Amarelas, pardas e indígenas	201.218	-4,6 (-12,9; -0,2)	0,040	2012-2016	4,9 (2,4; 11,9)	0,002	2016-2022
Ignorados	165.297	-13,0 (-19,6; -2,1)	0,010	2012-2014	-1,7 (-8,2; 7,5)	0,605	2014-2022
Motivo da internação							
Transtornos mentais e comportamentais devido ao uso de álcool	32.603	-8,1 (-14,8; -4,7)	<0,001	2012-2016	-1,4 (-3,4; 4,6)	0,527	2016-2022
Transtornos mentais e comportamentais devido ao uso de outras substâncias psicoativas	88.929	-11,2 (-24,8; 0,2)	0,056	2012-2015	3,0 (-3,0; 19,6)	0,132	2015-2022
Esquizofrenia, transtornos esquizotípicos e transtornos delirantes	215.034	-8,1 (-14,9; -4,6)	<0,001	2012-2016	-0,5 (-0,5; 4,9)	0,865	2016-2022
Transtorno de humor afetivo	253.907	1,7 (-0,1; 3,6)	0,071	2012-2022	-	-	-
Transtornos neuróticos, relacionados ao "stress" e somatoformes	12.496	-18,0 (-29,4; 5,1)	0,174	2012-2014	6,8 (-3,0; 28,0)	0,056	2014-2022
Outros transtornos	55.293	2,2 (0,4; 4,6)	0,020	2012-2022	-	-	-

a variação percentual anual da taxa no primeiro segmento temporal identificado pelo modelo Joinpoint; ^b^ variação percentual anual da taxa no segmento temporal subsequente identificado pelo modelo Joinpoint, quando aplicável.

**Figura 1:** Taxas de internações por questões psiquiátricas a cada 100 mil mulheres em idade fértil segundo as macrorregiões. Brasil, 2012-2022 (n=677.608)

## Discussão

Os resultados deste estudo mostram mudanças importantes nas internações psiquiátricas entre mulheres em idade fértil no Brasil na última década (2012-2022). O comportamento decrescente observado entre 2012 e 2016, seguido de estabilidade de 2016 a 2022, parece refletir transformações estruturais da rede de atenção psicossocial. A ampliação dos Centro de Atenção Psicossociais, o fortalecimento inicial das ações de saúde mental na atenção primária e o incentivo ao cuidado territorializado podem ter contribuído para inicialmente reduzir as internações em 2012-2016. No entanto, a estabilização nos anos seguintes (2016-2022) sugere possíveis limites desse avanço, sobretudo diante do subfinanciamento do SUS, das descontinuidades nas políticas de saúde mental e da reintrodução gradativa de práticas hospitalocêntricas^11^. Esses fatores, somados, podem ter tensionado a capacidade resolutiva dos serviços comunitários e gerado um cenário de demanda crescente pouco absorvida pela rede extra-hospitalar.

Diante dos achados observados, este estudo apresenta limitações inerentes ao uso de dados secundários, como subnotificação, inconsistências nos registros e ausência de padronização diagnóstica. Importa destacar que o padrão de internação caracterizado em nosso estudo não reflete o perfil psiquiátrico da população brasileira, uma vez que nossas análises se restringiram a mulheres em idade fértil. Ademais, as taxas de internação refletem oferta de serviços e fluxos locais, não prevalência populacional, o que restringe a extrapolação dos achados. Outro aspecto relevante a ser considerado é a diversidade socioeconômica e cultural entre as macrorregiões brasileiras, que resulta em variações nos protocolos de internação e heterogeneidade nos critérios diagnósticos, fatores que podem limitar a análise da verdadeira magnitude dos transtornos mentais e comportamentais na população. 

Mesmo assim, nossos resultados aportam elementos importantes para o planejamento em saúde mental. As diferenças observadas entre regiões, faixas etárias, raça/cor da pele e causas de internação reforçam desigualdades persistentes e apontam fragilidades na detecção precoce e na continuidade do cuidado no território.

As diferenças regionais observadas em nosso estudo seguem um padrão coerente com a literatura: as regiões Sul e Sudeste apresentam maior número absoluto de internações, o que pode estar relacionado tanto à maior prevalência de transtornos mentais em regiões urbanizadas - marcadas por violência, desigualdade e estressores econômicos - quanto à maior disponibilidade de serviços especializados, o que favorece maior registro e acesso^12^. A região Nordeste, em contraponto, apresentou uma inflexão nos números de internações a partir de 2014, com aumento considerável desses eventos. Esse achado pode estar associado a fragilidades e retrocessos na Política Nacional de Saúde Mental, no contexto da implementação da política de desinstitucionalização da rede de atenção psicossocial que ocorreu no mesmo ano. Esse recuo contribuiu para uma maior utilização de leitos psiquiátricos em unidades hospitalares^13^.

As internações foram mais frequentes (40%) entre mulheres brancas no total nacional, porém, padrões distintos emergem quando analisamos por macrorregião. No Norte, Nordeste e Centro-Oeste, para o período 2009-2018, mulheres pardas, pretas, amarelas e indígenas representaram a maior proporção (60%) das internações, evidenciando desigualdades estruturais e discrepâncias no acesso ao cuidado^14^. Ainda que essas regiões concentrem as maiores proporções (79%, 73% e 63%, respectivamente, segundo o Censo 2022) desses grupos raciais na população brasileira^8^, esses achados reforçam a presença do racismo institucional nos serviços, o que prejudica o acesso ao cuidado, a busca ativa e a continuidade do tratamento entre mulheres negras^14^^,^^15^. Soma-se a isso a incompletude de registros de raça/cor nessas regiões, o que prejudica a análise precisa da distribuição racial e dificulta o desenvolvimento de políticas de saúde pública eficazes. Esse conjunto de barreiras pode levar ao agravamento dos quadros clínicos e, consequentemente, ao maior risco de internações, especialmente em regiões de menor acesso a serviços especializados.

Em relação à idade, os resultados deste estudo revelam que, embora as internações sejam mais observadas entre mulheres de 30-39 anos, o crescimento mais expressivo ocorreu na faixa de 15-19 anos. Na última década, observou-se que adolescentes no mundo todo, de ambos os sexos, estão mais expostos ao estresse escolar, às pressões socioculturais e a mudanças diagnósticas que podem elevar a identificação de transtornos mentais^16^^,^^17^. Na Espanha (2000-2021) e na Itália (2016-2023), registrou-se aumento de internações por transtornos de humor em adolescentes desde as últimas duas décadas^17^^,^^18^. Esse padrão também foi observado no contexto brasileiro, como visto neste estudo. 

Na Finlândia, de 1996 a 2017, identificou-se que alterações hormonais relacionadas ao declínio do estrogênio durante a perimenopausa, entre mulheres a partir dos 30 anos, podem explicar a maior gravidade de sintomas e recidivas em transtornos psicóticos, favorecendo internações mais frequentes^19^. Nos Estados Unidos (2023-2024), foi observado que, embora geralmente se inicie após os 40 anos, pode ocorrer mais precocemente em algumas mulheres^20^. No Brasil, segundo Censo de 2022, essa distribuição das internações pode refletir a estrutura etária da população brasileira, na qual a maior proporção de mulheres (8%) se encontra na faixa etária de 35-39 anos^8^. 

A concentração de internações nessa faixa etária vista em nossos resultados reafirma o impacto dos fatores psicossociais em conseguir conciliar responsabilidades laborais, afetivas e reprodutivas. A alta demanda familiar e profissional repercute sobremaneira na saúde mental das mulheres, mas principalmente naquelas que têm ente 35 e 39 anos^19^^,^^20^. 

Quanto aos motivos de internação, os transtornos de humor (37%) e esquizofrenia e transtornos relacionados (32%) predominaram em todo o período analisado neste estudo. As regiões Norte (52 casos por 100 mil habitantes) e Nordeste (169 casos por 100 mil habitantes) apresentaram maiores taxas de internações por quadros psicóticos, o que pode ser resultado da associação entre pobreza e desigualdade social a maior risco e gravidade desses transtornos^21^^-^^23^. Parte dessas internações pode estar associada ao fenômeno de "porta giratória" marcado por reinternações frequentes em decorrência da fragilidade da atenção continuada, baixa adesão terapêutica e dificuldades no acompanhamento territorial^24^^-^^26^. Observou-se, também, aumento das internações por transtornos relacionados ao uso de substâncias, especialmente álcool e drogas ilícitas, indicando mudanças no perfil epidemiológico e um padrão de crescimento dessas internações em todas as regiões^17^^,^^27^.

Por fim, o aumento das internações entre adolescentes e a predominância de quadros psicóticos ajudam a destacar a necessidade de fortalecimento das redes de atenção psicossocial, ampliação do cuidado psicossocial e organização de estratégias mais efetivas de prevenção e manejo de crises, sobretudo para mulheres em idade fértil - um grupo muitas vezes invisibilizado nas políticas de saúde mental. Os resultados deste estudo fornecem subsídios importantes para o planejamento e a gestão de serviços de saúde do SUS, corroborando a necessidade de abordagem individualizada e direcionada ao cuidado à saúde mental de mulheres em idade fértil.

## Data Availability

Os dados utilizados neste estudo derivam de informações públicas disponíveis no repositório do Departamento de Informática do Sistema Único de Saúde, na base de dados de Morbidade Hospitalar do Sistema Único de Saúde - Sistema de Informações Hospitalares, disponíveis no Sistema de Tabulação de Dados: [http://tabnet.datasus. gov.br/cgi/deftohtm.exe?sih/cnv/nibr.def]. O conjunto de dados já tratado e utilizado para análise, bem como os códigos utilizados no processamento, podem ser disponibilizados mediante solicitação ao autor correspondente.

## Material Suplementar

Tabelas Suplementares
